# A Self-Organising Model of Thermoregulatory Huddling

**DOI:** 10.1371/journal.pcbi.1004283

**Published:** 2015-09-03

**Authors:** Jonathan Glancy, Roderich Groß, James V. Stone, Stuart P. Wilson

**Affiliations:** 1 Sheffield Robotics, The University of Sheffield, Sheffield, United Kingdom; 2 Department of Psychology, The University of Sheffield, Sheffield, United Kingdom; 3 Department of Automatic Control and Systems Engineering, The University of Sheffield, Sheffield, United Kingdom; UC Davies, UNITED STATES

## Abstract

Endotherms such as rats and mice huddle together to keep warm. The huddle is considered to be an example of a self-organising system, because complex properties of the collective group behaviour are thought to emerge spontaneously through simple interactions between individuals. Groups of rodent pups display two such emergent properties. First, huddling undergoes a ‘phase transition’, such that pups start to aggregate rapidly as the temperature of the environment falls below a critical temperature. Second, the huddle maintains a constant ‘pup flow’, where cooler pups at the periphery continually displace warmer pups at the centre. We set out to test whether these complex group behaviours can emerge spontaneously from local interactions between individuals. We designed a model using a minimal set of assumptions about how individual pups interact, by simply turning towards heat sources, and show in computer simulations that the model reproduces the first emergent property—the phase transition. However, this minimal model tends to produce an unnatural behaviour where several smaller aggregates emerge rather than one large huddle. We found that an extension of the minimal model to include heat exchange between pups allows the group to maintain one large huddle but eradicates the phase transition, whereas inclusion of an additional homeostatic term recovers the phase transition for large huddles. As an unanticipated consequence, the extended model also naturally gave rise to the second observed emergent property—a continuous pup flow. The model therefore serves as a minimal description of huddling as a self-organising system, and as an existence proof that group-level huddling dynamics emerge spontaneously through simple interactions between individuals. We derive a specific testable prediction: Increasing the capacity of the individual to generate or conserve heat will increase the range of ambient temperatures over which adaptive thermoregulatory huddling will emerge.

## Author Summary

Maintaining a constant body temperature is crucial to the survival of many species, including mammals such as rodents. Temperature regulation can be achieved via internal physiological processes and by seeking warmth when cold or cold when hot, as, for example, when young rats and mice exchange body heat by huddling together. Huddling is considered by many to be an important example of self-organisation, where a complex and adaptive group behaviour emerges from simple interactions between individuals behaving without plan or instruction. However, it is difficult to rule out the possibility that a complex group behaviour is the result of equally complex individual behaviours. Our approach is therefore to determine the simplest set of rules of interaction from which group-level huddling can emerge. Recent experiments have shown that huddling switches on at low temperatures, and that individuals continually exchange positions from the cool periphery of the huddle to its warm core. We show in computer simulations that both group behaviours emerge spontaneously when individuals simply turn towards sources of preferred temperature whilst continually generating, losing, and exchanging heat. A mathematical model of the simulation results suggests that individuals together behave as a single organism that continually adapts its exposed surface area to regulate its temperature in a way that no individual can. We therefore suggest that thermoregulatory huddling is a true self-organising system, and we derive from the model specific predictions that will enable future experiments to test this theory.

## Introduction

Many species of mammals [1, 2, 3], and birds [4, 5], spend a large proportion of their lives in direct contact with conspecifics, engaging in a synergistic pushing, climbing, wriggling, and burrowing behaviour referred to as ‘huddling’ [1]. For rodents, huddling begins at birth, when the dam first gathers her litter of around a dozen pups into a single aggregation, and it persists as the frequency and duration of her excursions from the nest increase [6]. Pups aged between 2 and 10 postnatal days reliably orient themselves in the direction of contact with a littermate [7], and pups that are displaced from the huddle center orient themselves back towards its center [1], suggesting that individual behaviours actively help to maintain the integrity of the huddle [8, 9].

In turn, the huddle is thought to help individuals to maintain their body temperatures [10, 11]. Compared with the adult, the metabolism of the neonate generates less heat, its lack of insulative fur and subcutaneous fat increases the rate of heat loss, and a higher surface area to volume ratio further limits the ability of the individual pup to thermoregulate [12, 13, 14], to the extent that pups are often considered to be *ectothermic* [4] (i.e., dependent on environmental heat sources). However, the metabolic rate of individual pups decreases as the number of huddling littermates increases [8, 15], and huddling slows the rate of heat loss from individuals by reducing their cold-exposed surface areas. Moreover, the exposed surface area of the entire litter has been observed to increase or decrease to adapt to the ambient temperature [1]. Hence it has been suggested that the litter of huddling neonates together behave like a single organism, which displays an *endothermic* thermoregulatory profile comparable to that of the adult [16].

**Fig 1:**
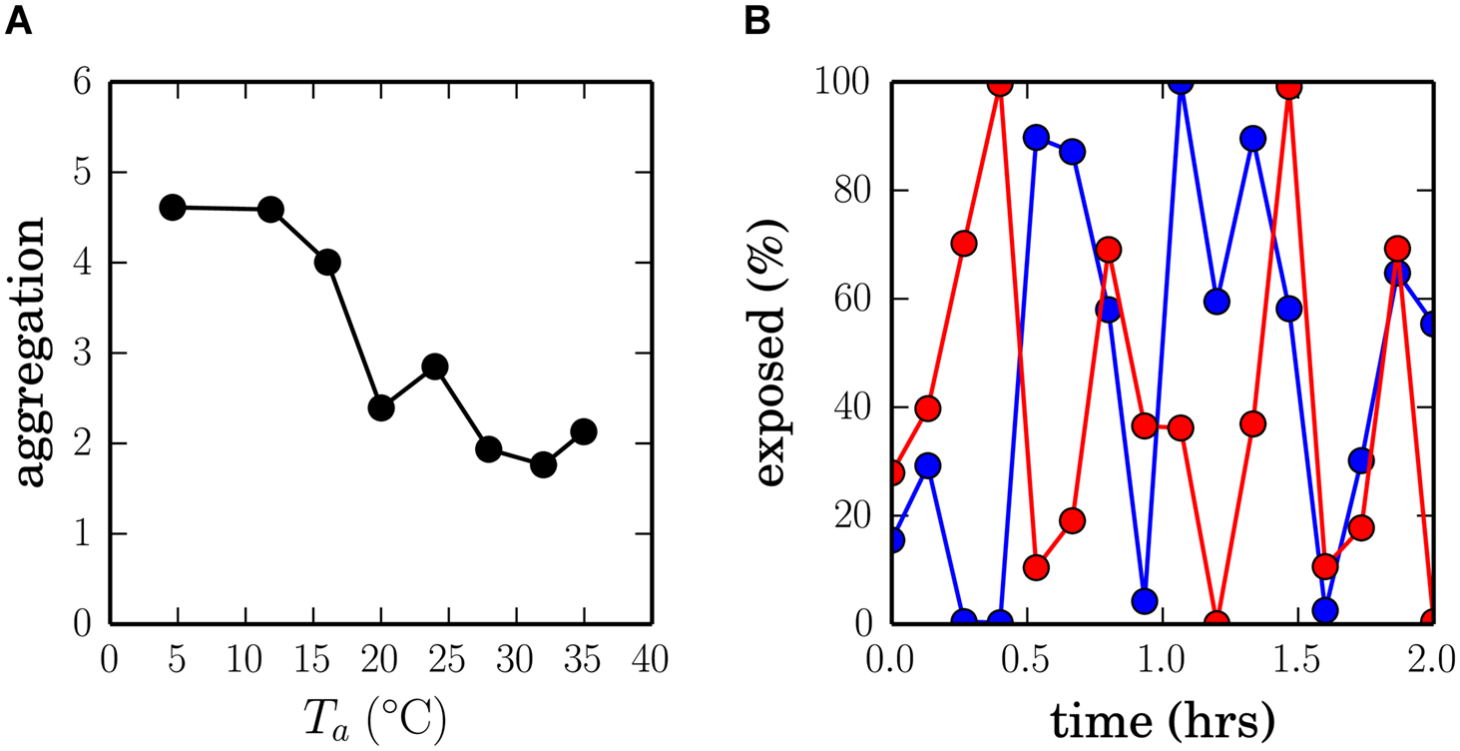
Huddling dynamics revealed by previous animal behavioural experiments. (A) Phase transition. Aggregation patterns in juvenile mouse litters were measured in experiments in which the ambient temperature *T*_*a*_ was experimentally manipulated [9]. In this study ‘aggregation’ was defined as the mean-variance coefficient of the number of individuals occupying cells of a grid overlaid on video frames from recordings of mouse litters (note that by this metric, an aggregation score of 1 is baseline). The data reveal what has been termed a second-order phase transition into huddling at low ambient temperatures, such that the litter huddle together when it is cold and disperse in a large arena when it is warm, with a smooth transition around a critical temperature in the range 15–25°C. (B) Pup flow. The proportion of time spent exposed at the periphery of an aggregation is shown for two focal pups from the same huddle, and varies periodically as individuals continually exchange positions between the cool periphery and the warm center. Data are reproduced, respectively, from [9] (original error bars removed and axes relabelled), and [17] (data from two pups collected into the same figure for convenience).

Behavioural experiments with rats and mice have identified two characteristic patterns in the dynamics of the huddle that could further improve thermoregulation. First, as the ambient temperature drops below a critical value, the dynamics of the huddle undergo what has been described as a second-order critical *phase transition* [9], i.e., an abrupt but continuous change in the degree of huddling. At high ambient temperatures the group dissipates, whereas at low ambient temperatures large aggregations of pups tend to form. Second, around the critical ambient temperature, huddling pups have been observed to continually exchange positions relative to the centre of mass of the huddle, in dynamics referred to as *pup flow* [1], which ensure that cooler pups at the periphery replace warmer pups at the centre [8], and which minimises the overall metabolic cost to all littermates [15, 9]. An illustration of the phase transition and the pup flow is provided in Fig 1.

Observations of the phase transition and pup flow have led many to consider the huddle to be a self-organising system, with adaptive thermoregulatory properties that emerge spontaneously from simple, local interactions between individuals, in the absence of any global supervisory mechanism (e.g., [1, 16, 9]). This view is supported by evidence from computational modelling studies showing how groups of agents evaluating only local rules of interaction can form and maintain a single aggregation. For example, the seminal model of Schank and Alberts (1997) [16] (see also [17]) shows how group-level aggregation patterns can be formed by simple agents making probabilistic decisions to navigate a grid-world, based on responding to obstacles detected in adjacent grid locations [16]. However, this model does not explicitly represent the heat exchanged between individuals and so was not designed to explore potential relationships between self-organisation of aggregate movement patterns and self-organisation of collective thermoregulatory dynamics. Conversely, a model proposed recently by Waters, Blanchette & Kim (2012) [18] provides a parsimonious account of thermoregulation via huddling, but this model represents the assumption that global supervisory mechanisms are in place to identify amongst the group both the coolest individual and the warmest location. Hence, to the best of our knowledge, no previous model of huddling has used only simple local interactions to govern individual behaviour *and* has explicitly represented the exchange of heat between individuals, and therefore previous models have not addressed either the emergence of a temperature-mediated phase transition or the emergence of a thermoregulatory pup flow.

The aim of the current paper is to determine whether the observed patterns of group contact and the group-level dynamics of heat exchange, could in principle both emerge via self-organisation. We present a simple self-organising model of thermoregulatory huddling that can explain each of these observations as emergent properties of the collective interactions of individuals. From the model we derive specific predictions that can be used to test self-organisation as a theory of thermoregulatory huddling.

## Results

### Thermotaxic Individuals

**Fig 2:**
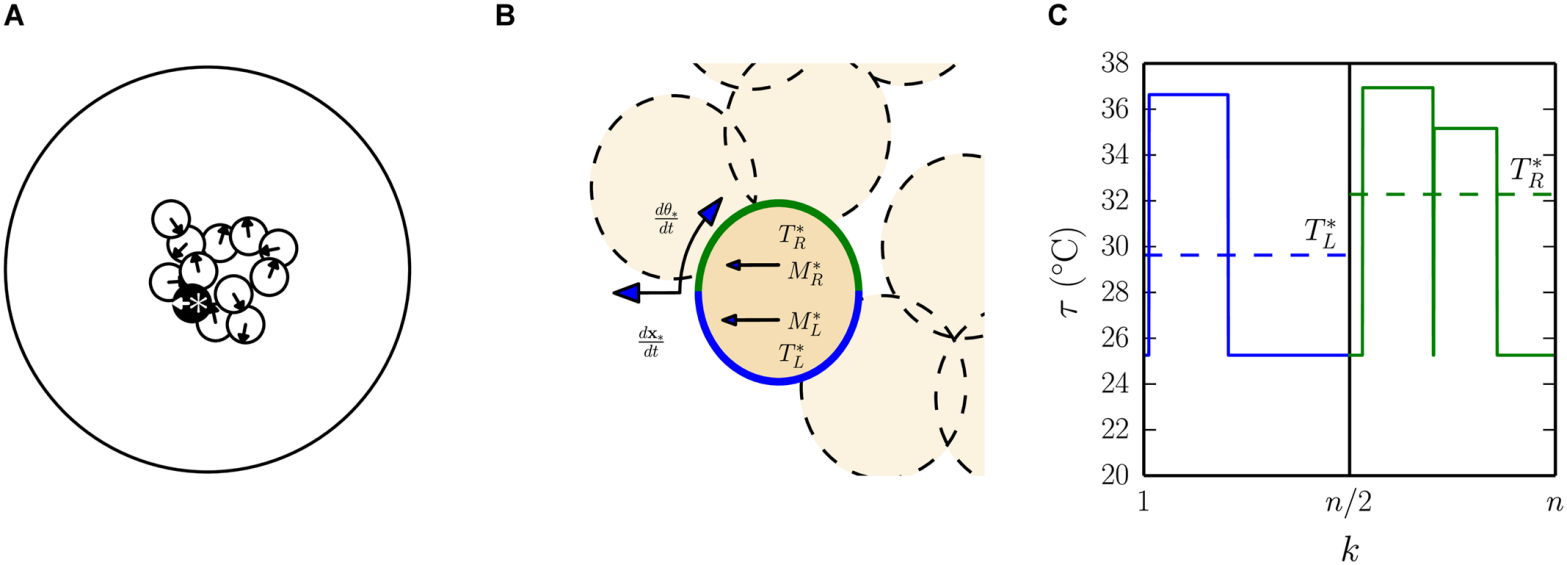
Modelling thermotaxic individuals. (A) A snapshot of the model, showing twelve simulated pups (small circles) in a circular arena (large circle), with orientations indicated by arrows. In this snapshot, pups are shown aggregated, often overlapping, and a focal pup is highlighted by a black body and a white *. (B) The same snapshot is shown, zoomed in on the focal pup. The left and right sides of its body are coloured green and blue respectively, to indicate the regions of the body surface over which average temperatures constitute the left and right sensor values $T_{L}^{*}$ and $T_{R}^{*}$. To implement thermotaxic control, these sensor values set the drive speed of contralateral motors $M_{L}^{*}$ and $M_{R}^{*}$, which change the position **x** and orientation *θ* of the pup. (C) For the focal pup, the temperature (*τ*) registered at discrete positions around the body circumference (indexed by *k*) is shown. For the focal pup $T_{R}^{*}$ is greater than $T_{L}^{*}$, indicating that it is warmer on the right than the left, hence at this point in time $M_{L}^{*}>M_{R}^{*}$ and therefore the pup will orient clockwise. See Models for precise definitions of these terms.

We set out to test whether the thermoregulatory properties of huddling observed in juvenile rodents could be explained as a product of self-organisation via simple, local interactions between individuals, in the absence of global supervisory mechanisms. We constructed an agent-based model, in which each pup is represented as a circle with 1000 thermal sensors (henceforth ‘thermometers’) evenly spaced around its circumference, that moves under simple thermotaxic control, orienting and moving towards sources of heat in a two-dimensional arena (see Fig 2). We hypothesised that this simple thermotaxic controller could be sufficient to reproduce the phase transition into huddling at low ambient temperatures, as observed in experiments [9, 1] in which litters were, in independent conditions, exposed to a range of constant ambient temperatures.

In the following sections we present a series of incremental modifications to our basic model of the pup as a thermotaxic ‘vehicle’ [19] to investigate the interplay between behavioural and physiological thermoregulation and the contribution of each to the collective behaviour. As we are modelling the litter as a (potentially) self-organising system, involving only individual-level rules of interaction, our approach necessarily involves modifying the description of the behaviour and physiology of the individual before simulating interactions between a litter of many individuals and analysing the group-level effects (see [20]).

In our basic thermotaxic simulation, each pup is modelled as a moving circular body with all thermometers on a given half of the circular body surface projecting to one of two ‘sensors’ (left or right). On each iteration of the simulation, the movement and orientation of each pup in a large arena with a circular boundary is computed following five steps; i) the left and right sensor values are determined by averaging the temperature registered by thermometers on either half of the body surface; ii) to generate thermotaxic orienting behaviours, the two resulting sensor values for each pup are used to determine the speed of a motor driving the opposite side of the body [19], such that a greater difference in temperature between the two halves of the body will cause pups to turn faster towards the warmer side; iii) the orientation and position of each pup is updated based on the motor speeds; iv) collisions between pups are resolved by making contacting pups spring away from each other with a force that increases with the degree of overlap between them; and v) the body temperature of each pup is updated based on the temperature and proportion of thermometers that are either exposed to the ambient temperature or in contact with another pup. Equations describing these steps in full are provided in Models. Note that we allow no distal sensing of either proximity or temperature, hence our modelling approach is to assume that information is exchanged only locally, between contacting pups.

### Endothermic Individuals

The key assumption represented by our first and simplest model is that individual pups are able to maintain a constant body temperature of *T*_*b*_ = 37°C. Hence in this model, step v of the algorithm described above is redundant. Accordingly, at each step of the simulation, each thermometer of each pup detects either the ambient environmental temperature or the 37°C body temperature of any pup with which it makes contact. Reflecting the perfect capability of each individual to maintain a constant body temperature, we refer to this as the endothermic individuals (or just the endothermic) model. This model represents the minimal set of constraints that we anticipated *a priori* could account for the phase transition reported by Canals et al., (2011) [9]. Some preliminary experiments with this model are presented in [21].

By analogy with the experiment of [9] we simulated the endothermic model at twenty ambient temperatures *T*_*a*_ ranging from 5°C to 50°C at regular intervals. Each simulation consisted of 8,000 time-steps (iterations of steps i-v, as described above), and analyses were carried out with respect to data averaged over ten replications, with the initial positions of each of twelve pups distributed uniform randomly within a distance of one pup radius from the center of the arena (pups are therefore close together at the onset of each simulation; see Discussion for justification).

We observed that at ambient temperatures above approximately 37°C, simulated pups oriented away from contacts and dissipated from their initial positions, whereas at lower ambient temperatures pups tended to quickly collect together into aggregations in which multiple pups continue to maintain contact for the duration of the simulation. This group-level behaviour is reasonably straightforward to intuit, because at low ambient temperatures pups will sense a higher average temperature on the side of the body where most contacts occur, which increases the relative drive speed of the contralateral motor, and results in the pup orienting towards contacts. Conversely, at ambient temperatures greater than 37°C the side with least contact will register the greater sensor value, and so pups will orient away from contact.

A simple thermotaxic scheme is therefore sufficient to reproduce the phase transition into huddling at low ambient temperatures observed experimentally, at least in terms of our group-level metric of huddling, which we define to be one minus the proportion of exposed thermometers (1 − *η*; see Models), averaged across pups and simulation time-steps. Ambient temperatures above 37°C lead to values of 1 − *η* ≈ 0.2 (i.e., 80% thermometers exposed), whereas ambient temperatures below 37°C lead to 1 − *η* ≈ 0.5, either side of a steep transition at the critical 37°C body temperature. The overall trend in aggregation patterns predicted by this model is comparable to that measured by Canals et al. (2011; [9]; see Fig 1).

**Fig 3:**
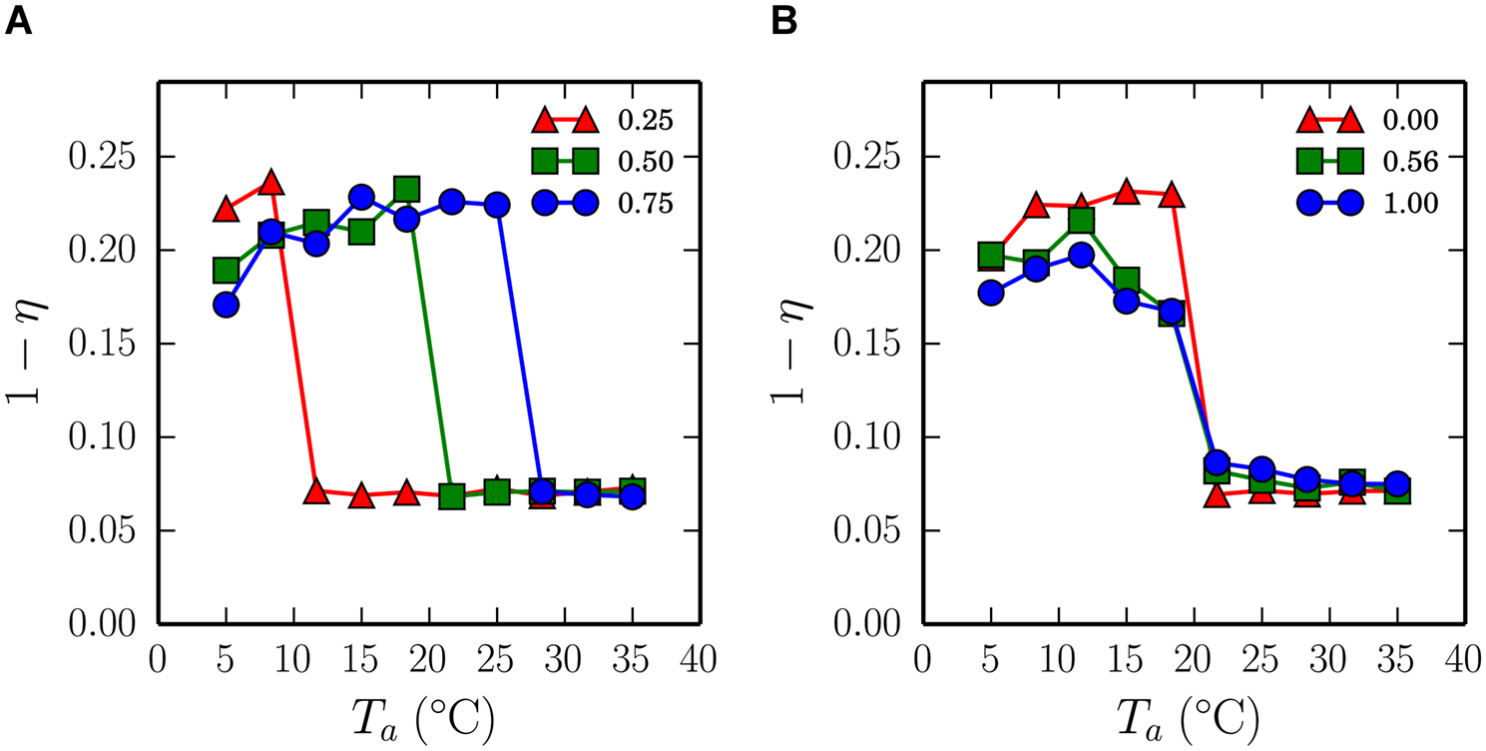
A phase transition emerges in simulations of the endothermic individuals model. Simulation of the experiment of [9], in which each pup simply turns in the direction of heat sources. The ordinate axis represents the mean proportion of pups’ body surfaces that are in contact with another pup, (1 − *η*) averaged across pups and time-steps within a simulation and across 10 repeated experiments with random initial conditions. (A) The critical temperature of the phase transition can be increased by arbitrarily scaling the temperature registered at each point of the pup body surface to be 0.25, 0.5, and 0.75 of the ambient temperature (legend denotes scaling factor). (B) With the temperature scaling set to 0.5, the slope of the phase transition can be smoothed to better match the form of the experimental data presented in Fig 1, by adding normally distributed noise to the temperature sensed at each point on the pup body, with variances 0.0, 0.56 and 1°C (indicated by the legend) increasing the smoothness of the transition. Tuning the endothermic model in this way can give a reasonable match to the experimental data but it generates qualitatively poor huddling, as explained in the main text.

However, closer inspection of the simulation and animal data reveals two important differences. First the critical temperature at which huddling ‘switches on’ is around 20°C for the animals, which is lower than the prediction of the model that the critical temperature should be the 37°C body temperature. Second, the form of the transition predicted by the model is more similar to a Heaviside step function than to the smoother sigmoidal shape of the transition in the animal data. It is simple to manipulate the model to account for these differences. For example, we can lower the critical temperature of the transition by setting the heat registered by each thermometer to be an arbitrary fraction of the body temperature of contacting pups, and we can likewise add noise to all thermometers at each simulation time-step to smooth the transition. Fig 3 shows how these manipulations can be used to create a good fit of the model to the experimental data. However, these improvements in terms of our group-level huddling metric mask a more important weakness of the endothermic individuals model.

Whilst the endothermic model quantitatively reproduces the phase transition at the macro-level, visual inspection of the aggregation patterns formed in each simulation revealed a strong tendency for the initial aggregation of pups to fracture into several smaller isolated groups, rather than to maintain one global cluster comprising all pups. We refer to the small isolated clusters as ‘micro-huddles’, and to the global aggregation typically observed in animal experiments as a ‘macro-huddle’. Although micro-huddles have been observed experimentally, they are not typical at low ambient temperatures [17, 9]; hence the endothermic model is unable to account for the maintenance of naturalistic aggregation patterns.

### Ectothermic Individuals

In the endothermic model, all pups were assumed to be capable of maintaining a constant body temperature, *T*_*b*_ = 37°C. We observed that a single pre-formed large macro-huddle fractured into small micro-huddles. Hence we interpret micro-huddles as stable local maxima and macro-huddling as an unstable global maximum solution to the collective thermotaxic dynamics of the system. We reasoned that the global solution could be stabilised if individuals could be attracted towards heat propagating from more distal pups via a chain of intermediaries. Supporting the propagation of heat along a chain of contacting pups requires the body temperature of each pup to be capable of changing over time.

In contrast to the assumption of the endothermic model, and in contrast to the thermoregulatory capacity of mature rodents, juveniles have only a weak capacity to regulate their body temperature, to the extent that pups are often described as ectothermic [17]. Hence, in what we call the ectothermic model, we allow the body temperature $T_{b}^{i}$ of each pup (indexed by *i*) to change over time. The following equation captures three key dynamics that we assume govern the change of the body temperature of the individual pup ($\frac{dT_{b}^{i}}{dt}$); i) heat decay, ii) heat exchange, and iii) heat generation:

$$\begin{array}{c} \frac{dT_{b}^{i}}{dt}=-k_{1}\eta_{i}\left(T_{b}^{i}-T_{a}\right)-k_{2}\left(1-\eta_{i}\right)\left(T_{b}^{i}-T_{c}^{i}\right)+G. \end{array}$$ (1)

The first term on the right of Eq 1 represents our assumption that pups continually lose heat to the environment, mediated by factors such as the amount of insulative fur and subcutaneous fat, which we collect together in the thermal conductance constant *k*_1_, and which we scale by the proportion *η* of thermometers that are exposed. The second term describes how heat is exchanged between pups that are in contact, mediated by a second thermal conductance constant *k*_2_. $T_{c}^{i}$ is the contact-mediated surface temperature; i.e., the sum temperature registered by the thermometers of pup *i* that are in contact with a littermate (see Models for a precise definition). The final term of Eq 1 represents the generation of a small, constant rate of heat by each pup via internal physiological processes, namely via the brown adipose tissue (BAT) thermogenesis system ([22]; see Discussion). Note that according to this model, for an isolated pup at thermal equilibrium (i.e., *η* = 1, and $\frac{dT_{b}^{i}}{dt}=0$), Eq 1 yields the *endothermic* relation often used to describe the metabolic rate of an individual, *G* = *k*_1_ *η*(*T*_*b*_−*T*_*a*_) ([23]; see also [24, 25, 9]).

We simulated the model as before, now iterating the ectothermic equation, Eq (1), at each time-step, and we found two important differences in the group-level behaviour.

First, we were able to confirm that the new thermodynamics are sufficient to perturb the system into reliably maintaining a macro-huddle. This observation was supported by high values of our huddling metric; around 0.6 in the majority of simulations. We observed that the emergent macro-huddles maintain a core of warmer pups surrounded by a periphery of cooler pups, which is consistent with the experimental observations of [26], for example. Moreover, we observed that individual pups tend to move constantly with respect to the center of mass of the huddle. These group-level dynamics suggest that a continual ‘pup flow’ as described by [1] might emerge from a model of this form, although we delay a formal analysis until the penultimate results section.

The second important difference is that in the ectothermic model the phase transition into huddling at low ambient temperatures ceases. Instead, stable macro-huddles emerge and persist for the duration of the majority of simulations, irrespective of the ambient temperature. The loss of the phase transition in the ectothermic model can be explained in terms of the thermogenesis term *G* > 0 in Eq 1, which ensures that the temperature of the body is always at least that of the environment in the steady state ($T_{b}\geq T_{a}+\frac{G}{k_{1}\eta }$), and which therefore determines that our simple thermotaxic pups will always orient towards another pup.

### Homeothermotaxic Individuals

To achieve a system capable of maintaining stable global huddles that dissipate at high ambient temperatures, we require a model in which each pup is able to display a preference for higher contact-meditated temperatures at cooler ambient temperatures, and for lower contact-mediated temperatures at warmer ambient temperatures. To this end, we note that when isolated on a thermocline (i.e., an approximately linear temperature gradient) rat pups will not climb the temperature gradient indefinitely towards the highest temperature, an implicit prediction of our ectothermic model, but instead they will move through the temperature gradient to a point that allows them to maintain a body temperature of 37°C with minimal metabolic cost [14]. Importantly, isolated pups show an ability to navigate both up and down a temperature gradient as required to achieve thermal homeostasis [27].

Naively, we can introduce a preferred temperature *T*_*p*_ into the model simply by changing the linear mapping of temperatures sensed at the body surface into motor drives (see Models) to instead be a non-monotonic function, e.g., a Gaussian, $e^{-\frac{{\left(T-T_{p}\right)}^{2}}{\sigma }}$, where *T* = *T*_*l*_+*T*_*r*_ is the sum of the temperature measured on the left and right of the body. Such a mapping would ensure that individuals display a temperature preference when isolated on a thermocline. However a model of this form cannot account for data showing that the temperature at which an individual pup will settle on a thermocline adapts to changes in its ability to generate heat. Farrell & Alberts (2007) [27] pharmaceutically manipulated thermogenesis in seven-day old rat pups using norepinephrine and found that they will move to a position on a thermocline that compensates for the resulting physiological change; pups generating more heat will settle at a (proportionately) cooler location than controls to maintain a constant 37°C body temperature.

Thus we introduce into the model not an explicit preferred environmental temperature, but rather a drive to move so as to reduce the discrepancy between the actual body temperature and *T*_*p*_ as a target body temperature, i.e., by introducing a function of the form,

$$\begin{array}{c} f\left(T\right)=\left(T_{p}-T_{b}\right)T. \end{array}$$ (2)

We implement the assumption represented by Eq 2 by adding to the drive of the left and right ‘motors’ of each pup, the sum of the body temperatures of the contacting pup that is nearest to each contralateral thermometer, before squashing the result with a steep (effectively linear) sigmoid to ensure that all motor drives are positive (see Models). The result of this modification is that the orienting of each pup brings *T*_*b*_ towards *T*_*p*_ by a form of gradient descent that allows for the body temperature of the individual to achieve homeostasis. Consequently, simulated pups isolated on a thermocline will settle to a location that maintains the body temperature at *T*_*p*_, so as to compensate for any variation in the rate of thermogenesis, *G*. We therefore refer to this as the homeothermotaxic individuals model.

**Fig 4:**
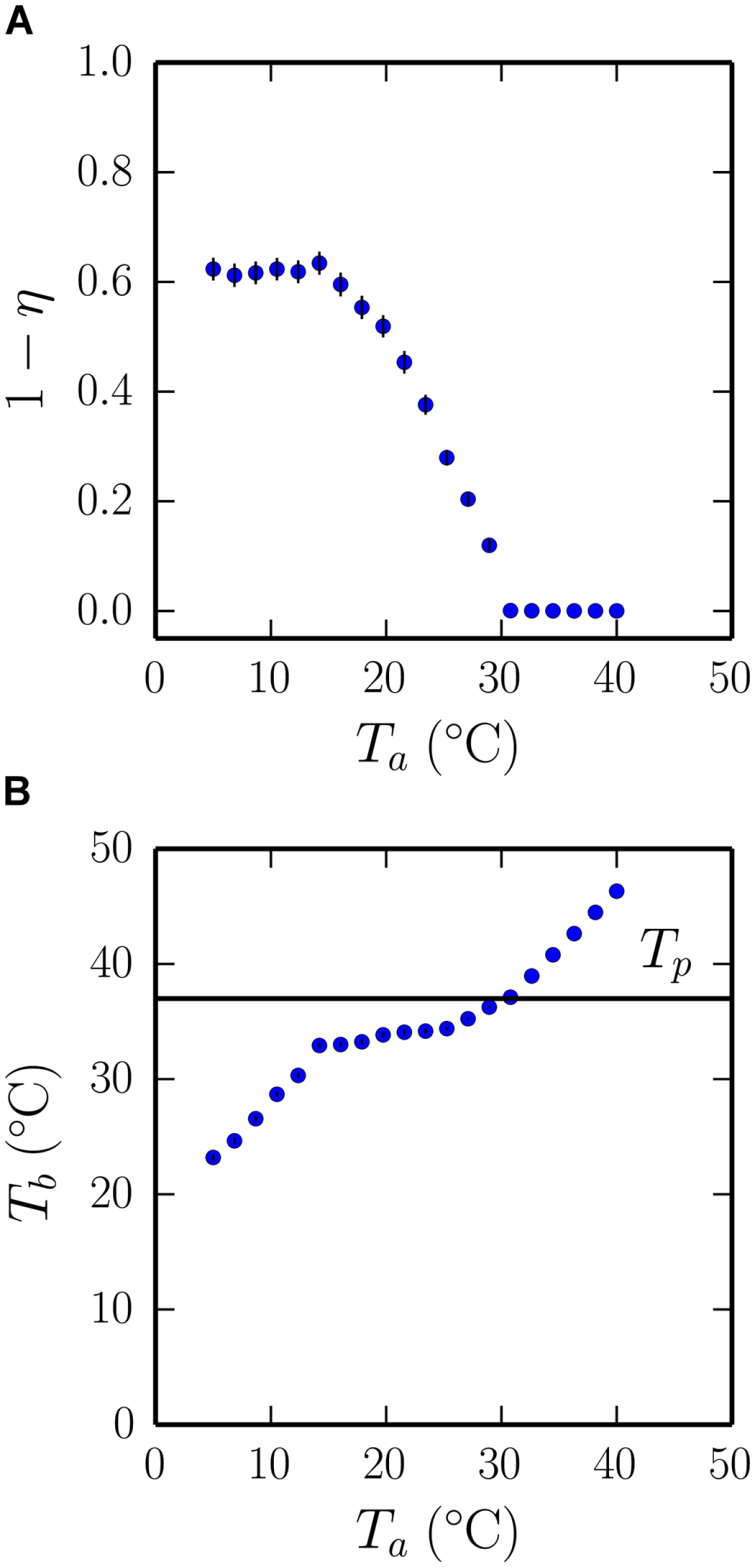
Thermoregulatory huddling in the homeothermotaxic individuals model. (A) The phase transition returns in the collective huddling behaviour (1 − *η*) of the full model. Here individual pups are ectothermic, generating their own heat which is dynamically exchanged between individuals and decays towards the ambient temperature *T*_*a*_, and orienting responses direct pups towards heat sources with which they make contact that bring them closer to their preferred 37°C body temperature *T*_*b*_. (B) The average body temperature is shown as a function of the ambient temperature, which reveals that for a range of temperatures the huddle is able to adaptively maintain a stable 37°C temperature (shown as a solid line). Hence, huddling in the model is thermoregulatory, enabling endothermic dynamics to emerge from local interactions within a group of ectothermic individuals. Data are averages of ten randomly seeded simulations in which the rate of thermogenesis *G* = 6.32 was chosen to give an approximate fit between panel A and the data presented in Fig 1. Error bars show standard error, calculated for 120 observations (ten simulations, each with twelve pups).

When we subject the homeothermotaxic model to the experimental protocol of [9] we observe that the phase transition reappears, and that macro-huddles emerge at low ambient temperatures. See Fig 4.

A key question that we have not yet addressed is to what extent can the emergent aggregation patterns benefit the individuals that collectively give rise to them? We therefore examined the average body temperature of the litter as a function of the ambient temperature, and identified three distinct regions that correspond with three distinct regions of the phase transition. As expected, for simulations at high ambient temperatures where pups tend to dissipate, and thus tend not to be in contact, the average body temperature of the litter varies precisely with the ambient temperature. However, for simulations at low ambient temperatures the average temperature of the litter becomes much higher than the ambient temperature, as individuals cluster to maintain the heat that they collectively generate. Hence, huddling at low ambient temperatures increases the average temperature of the litter, and confirms a group-level advantage to huddling at low ambient temperatures. Interestingly, for a range of ambient temperatures that corresponds to the slope of the huddling phase transition, the average body temperature remains approximately constant, which suggests that beyond simply warming the litter when it is cold, huddling helps to regulate body temperatures over a range of intermediate ambient temperatures.

### The Emergence of Pup Flow

We observed in our simulations of the homeothermotaxic model that for a range of ambient temperatures, around the critical temperature for the phase transition, pups appeared to continually exchange positions with respect to the centre of the macro-huddle. Pups in the center of the huddle will remain there until their body temperature rises above the preferred temperature, at which point they will move to the periphery. Similar dynamics observed in real litters at low ambient temperatures have been termed ‘pup flow’ [1].

We objectively quantify the degree of pup flow in terms of the average absolute value of the time derivative of the proportion of exposed surface area, i.e., $\frac{1}{n_{t}-1}\sum_{t=2}^{n_{t}}|\eta (t)-\eta (t-1)|$ for *n*_*t*_ simulation time-steps. This metric reflects the overall rate at which pups exchange positions with respect to the aggregations that they belong to. As a pup changes from being at the center of the huddle to being at the periphery it will contribute a positive time-difference in exposure, and when a pup changes from periphery to center it will contribute a negative difference, hence the absolute value indicates the total rate of flow. Pups that remain in the center or periphery of a huddle, or remain isolated, will have a constant degree of exposure and hence will not increase the pup flow metric.

**Fig 5:**
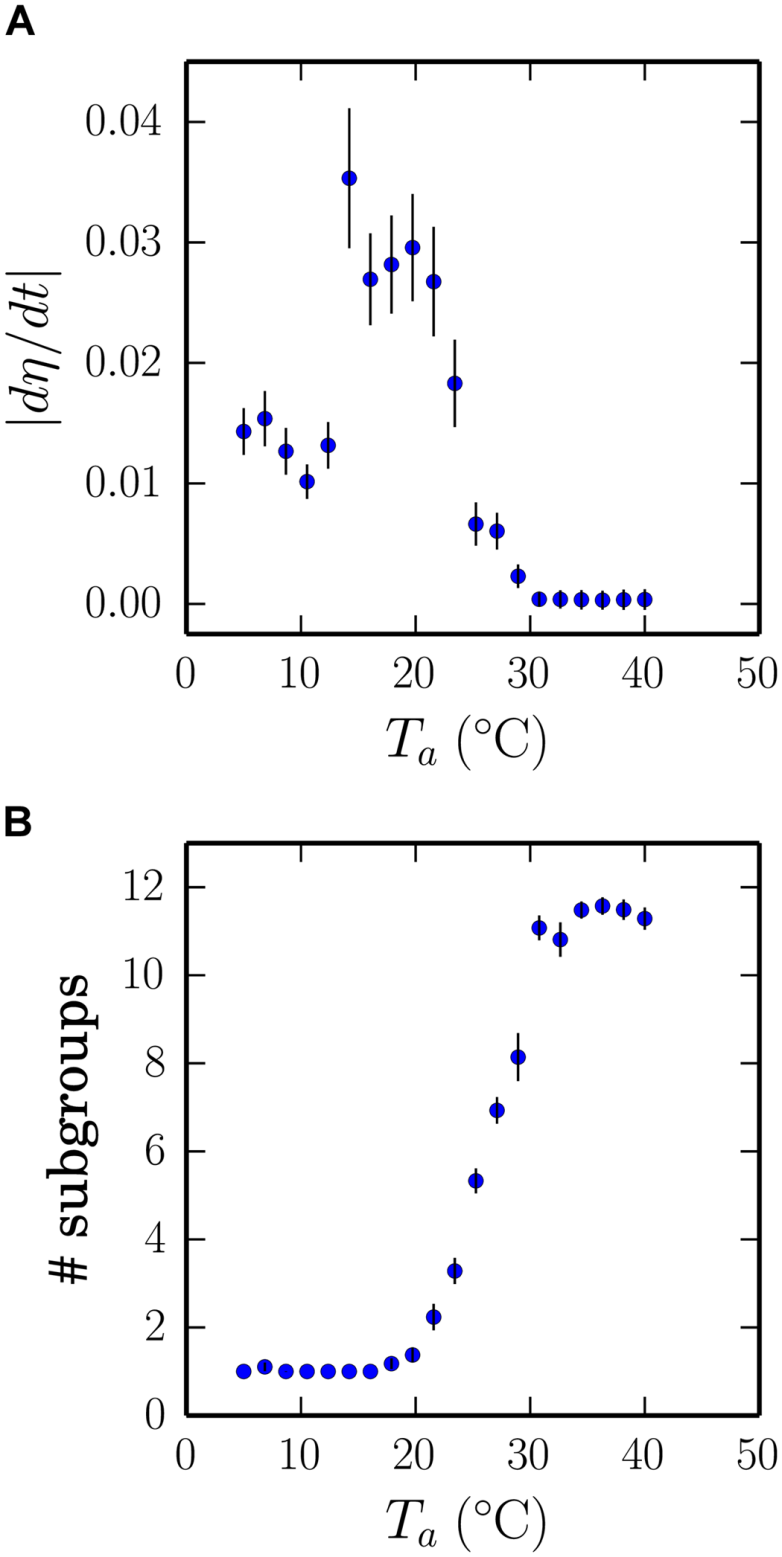
Quantifying pup flow. (A) Pup flow was quantified as the time-averaged absolute time-derivative of the exposed surface areas of the pups. The rate of pup flow was small at low and high ambient temperatures, but was large during the sloping region of the phase transition, where pups were observed to continually exchange positions between the center and periphery of macro-huddles or between micro-huddles. (B) To quantify aggregation level, we observed the average number of distinct groups that form at each ambient temperature. At low ambient temperatures a single macro-huddle is maintained, while as the temperature is increased beyond a critical point pups arrange into aggregation patterns with many smaller micro-huddles until eventually huddling ceases. Error bars in A show standard error, calculated for 120 observations (ten simulations, each with twelve pups) and in B for 10 observations.

Fig 5A shows how the pup flow varies across a range of ambient temperatures. Like the corresponding plots of huddling and average body temperature in Fig 4, pup flow varies in three distinct regions. At low ambient temperatures, when we observe that macro-huddles tend to be maintained, the mean absolute time derivative of *η* remains at around 0.013, reflecting a small but constant change in the configuration of the macro-huddle as it swells and contorts under the movement of the litter. As the ambient temperature is raised, and the slope of the huddling phase transition begins, there is a sharp rise in flow peaking at around *T*_*a*_ = 16°C where we observe that pups comprising a macro-huddle will constantly flow between the center and periphery. Based on observing the aggregation patterns as they unfold we identify the group dynamics in this initial peak to be qualitatively similar to the pup flow characterised by [1]. Simulations at higher ambient temperatures yield a reduction in pup flow, and here we observe that the flow is not maintained in a macro-huddle but instead reflects the exchange of pups between nearby micro-huddles. As the ambient temperature is raised further and the pups start to disperse, the peak in flow drops rapidly, approaching a zero baseline for *T*_*a*_ > 30°C where all pups tend to remain isolated.

Fig 5B shows how the average number of subgroups (micro-huddles) in our simulations varies with the ambient temperature. The relationship follows an approximately sigmoidal curve, with a linear zone corresponding to the range of temperatures in which we observe pups to be flowing between subgroup states (see Discussion and supplementary material S1 Text).

### The Huddle as a Super-Organism

We have seen that the homeothermotaxic individuals model allows the configuration of the group to adapt to temperature changes in the environment, such that at lower ambient temperatures a lower overall exposed surface area enables the group to conserve the heat generated by each pup, leading some authors to think of the huddle as a single organism (e.g., [8]). We therefore ask; if the group behaves as a single entity, optimising its overall exposed surface area, how would its dynamics compare to those of the full agent-based self-organising system? We answer analytically, by adapting our description of the individual into a description of an entire litter, as a ‘super-organism’ capable of modifying its exposed surface area. To this end we first remove the heat exchange term from Eq 1. Then to highlight the change in interpretation from an individual-level description to a group-level description we substitute the individual exposed surface area *η* from Eq 1 with a similar parameter *A* representing the overall exposed surface area of the super-organism. As we are interested in the settled temperature of the super-organism we define 0 = *k*_1_ *A*(*T*_*a*_−*T*_*b*_)+*G*, and to represent the assumption that the super-organism is able to maintain a preferred temperature, we set *T*_*b*_ = *T*_*p*_. The result can be rearranged to determine how the surface area of the super-organism should adapt so as to maintain thermal homeostasis: $A=\frac{G}{k_{1}(T_{p}-T_{a})}$. Finally we obtain *A*_min_ = 0.36 and *A*_max_ = 1.0 as the minimum and maximum values measured over all agent-based simulations with the homeothermotaxic model, and impose these same limits on the exposed surface area of our super-organism:

$$\begin{array}{c} A\left(T_{a}\right)=\{\begin{array}{cc} A_{\text{min}} & \text{if}\;T_{a}\leq T_{p}-\frac{G}{k_{1}A_{\text{min}}}\\ A_{\text{max}} & \text{if}\;T_{a}\geq T_{p}-\frac{G}{k_{1}A_{\text{max}}}\\ \frac{G}{k_{1}\left(T_{p}-T_{a}\right)} & \text{otherwise}. \end{array} \end{array}$$ (3)

For a direct comparison with the behaviour of the full agent-based model we define the huddling metric as 1 − *A*(*T*_*a*_). By the same logic we can derive a prediction from the super-organism model for how the mean body temperature *B* should vary with the ambient temperature:

$$\begin{array}{c} B\left(T_{a}\right)=\{\begin{array}{cc} T_{a}+\frac{G}{k_{1}A_{\text{min}}} & \text{if}\;T_{a}\leq T_{p}-\frac{G}{k_{1}A_{\text{min}}}\\ T_{a}+\frac{G}{k_{1}A_{\text{max}}} & \text{if}\;T_{a}\geq T_{p}-\frac{G}{k_{1}A_{\text{max}}}\\ T_{p} & \text{otherwise}. \end{array} \end{array}$$ (4)

**Fig 6:**
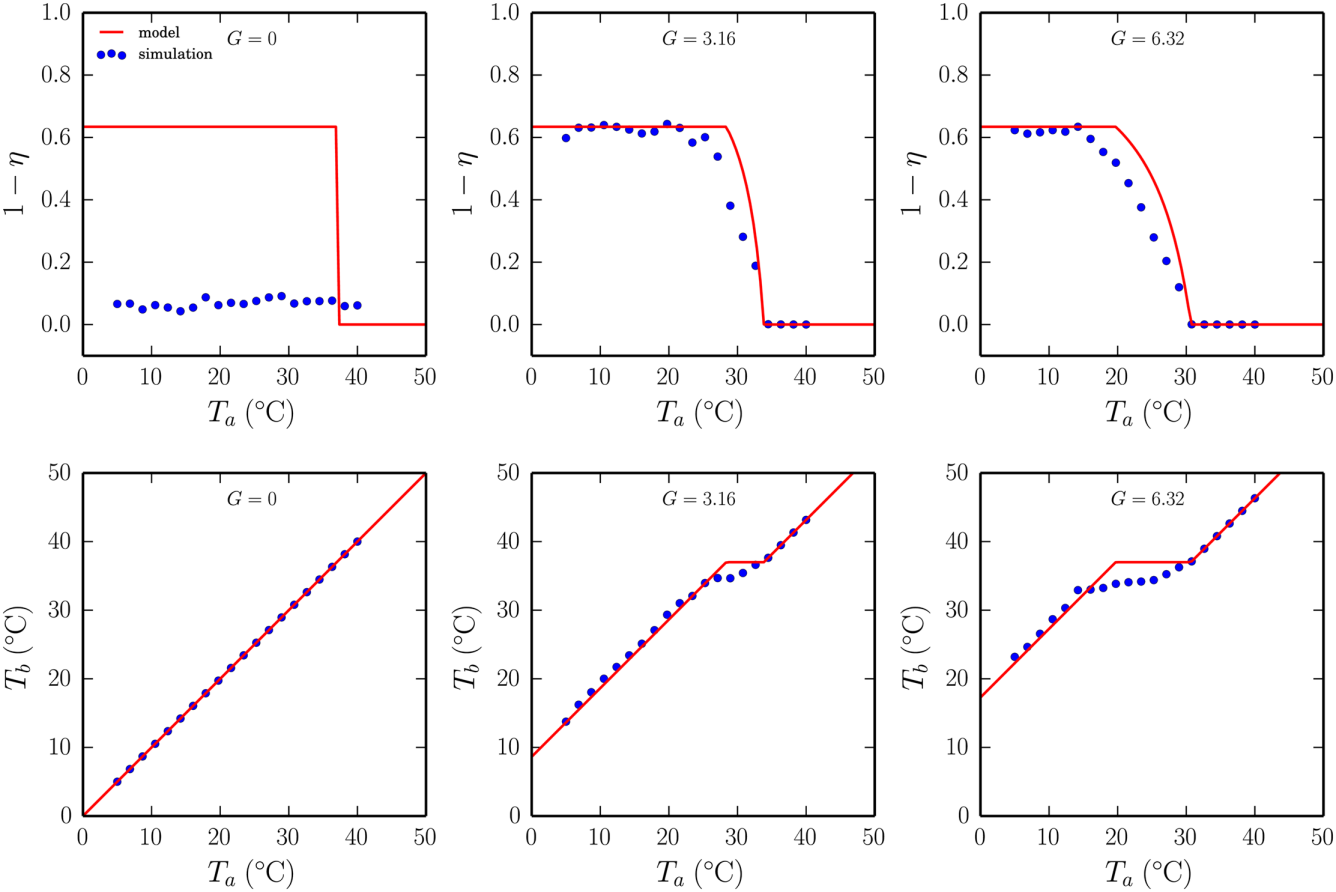
The huddle as a super-organism. The homeothermotaxic model was simulated for a range of rates of thermogenesis *G*. Plots of huddling are shown in the top row and corresponding plots of the average body temperature are shown in the bottom row. Increasing *G* smooths the huddling phase transition and increases the critical ambient temperature at which the transition occurs. The critical region of the phase transition corresponds to the range of ambient temperatures over which the average temperature of the litter is maintained at the preferred 37°C. We found a close agreement between the simulation data (filled circles) and that predicted by an analytical model (solid lines) that we derived by considering the huddle as a single super-organism with thermodynamics based on our ectothermic individuals model, with the additional capacity to adapt the overall exposed surface area of the group to maintain thermal homeostasis. The simulation and model data agree closely for all conditions, except where *G* = 0, where the model incorrectly predicts a sharp phase transition at the preferred temperature.

Fig 6 shows that the behaviour of the huddle, when considered as a single organism that adapts its exposed surface area to changes in the ambient temperature, is remarkably similar to that predicted by the agent-based (homeothermotaxic) model, with a notable exception when thermogenesis is absent. When *G* = 0 all agent-based simulations result in a dispersion of pups, irrespective of the ambient temperature, whereas the super-organism model predicts that strong huddling should persist until the environment is warmer than the target 37°C body temperature. In this case it is interesting that both the simulation and analytical models predict a linear relationship between the ambient and body temperatures, neither fully able to achieve thermal homeostasis, suggesting that huddling and non-huddling are to be considered equally valid thermoregulatory solutions in the absence of thermogenesis. We will return to this point in the Discussion.

Otherwise, a strong agreement between the results of the agent-based model and the model of group-level adaptation motivates an interpretation of the agent-based model as a unitary system that uses collective thermotaxis to adaptively control its overall exposed surface area in order to regulate its temperature. Hence these results support a view of the huddle as the collective expression of thermotaxis amongst individuals, from which a super-organism emerges with a thermoregulatory capability superior to that of the individual.

**Fig 7:**
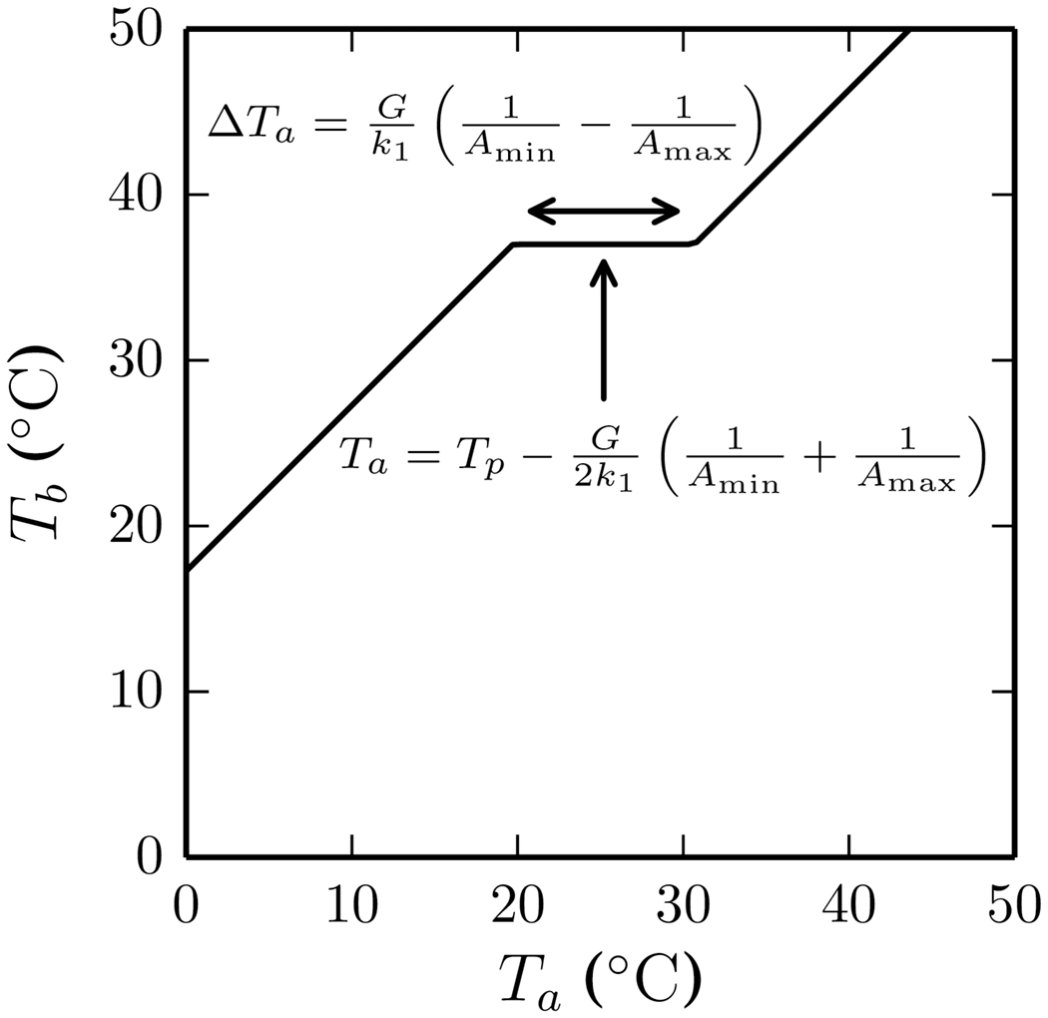
Testable predictions of the model. By approximating the huddle as a single super-organism, we are able to derive the following prediction from our theory of thermoregulatory huddling as the self-organising product of simple local interactions between pups. Accordingly the key parameter is the term $\frac{G}{k_{1}}$, where *G* is the rate of thermogenesis and *k*_1_ is the thermal conductance of each pup. The model predicts that either increasing thermogenesis (e.g., by pharmaceutically enhancing the action of brown adipose tissue) or decreasing the thermal conductance (e.g., by insulating each pup) will increase both the critical ambient temperature (single-headed arrow) and the range of ambient temperatures (double-headed arrow) over which the temperature of the huddle is stable. Confirming this prediction in future experiments would provide strong support for our description of the huddle as a self-organising system.

We can see from the super-organism model Eqs (3) and (4) that the region in which huddling behaviours keep body temperatures constant, which corresponds to the slope of the huddling phase transition and the plateau in Fig 6, extends across a range of ambient temperatures $\Delta T_{a}=\frac{G}{k_{1}}\left(\frac{1}{A_{\text{min}}}-\frac{1}{A_{\text{max}}}\right)$, that is centred on $T_{a}=T_{p}-\frac{G}{2k_{1}}\left(\frac{1}{A_{\text{min}}}+\frac{1}{A_{\text{max}}}\right)$. See the annotated sketch in Fig 7 for an illustration. Hence the central testable prediction of our model is that either pharmaceutically increasing thermogenesis *G* or insulating pups to reduce the thermal decay *k*_1_ will increase the set point and the range of temperatures over which body temperatures will be regulated via huddling behaviour. Failure to confirm these two hypotheses would falsify the theory represented by the model, that adaptive thermoregulatory huddling self-organises from simple local homeothermotaxic interactions between individuals.

## Discussion

We have presented an agent-based model of thermoregulatory huddling behaviours in juvenile rodents as a self-organising system, according to which individuals behave like the simple vehicles of Braitenberg’s thought experiments [19], orienting in the direction of heat sources. Our model adds support for the theory that both the aggregate patterns of group contact and the thermoregulatory properties of huddling can emerge via self-organisation from simple local interactions between animals.

According to the model, there are two requirements for the emergence of thermoregulatory huddling. First, the body heat of each pup needs to be dynamic, such that it continually decays to the ambient temperature, is exchanged with contacting pups, and is generated by each littermate. Second, each individual should orient towards sources of heat more similar to its preferred temperature than its current temperature. When these two mechanisms are in place, a sufficiently low ambient temperature will naturally trigger the emergence of an aggregation pattern. We identify this collective behaviour as thermoregulatory huddling on the grounds that it adaptively maintains the body temperatures of all individuals and produces a phase transition under experimental manipulation of the ambient temperature, and such that pups continually flow from the cool periphery to the warm center. These phenomena have been observed in real litters of rodents. The key feature of the model, that distinguishes it from other models of thermoregulatory huddling, is that these collective behaviours emerge from only local interactions between individual animals, in the absence of a global supervisor that accesses information about the state of multiple animals. We therefore interpret the results as evidence that thermoregulatory huddling in young rodents is the product of self-organisation.

Our model represents an extreme version of this theory, where thermo-tactile information is exchanged only when pups make contact. Hence the model serves as an existence-proof for the plausibility of the hypothesis that the known thermoregulatory properties of the huddle emerge via self-organisation, in addition to the self-organisation of aggregate patterns of group contact established by the model of [16]. We presented our model as a progression through a series of refinements to the underlying assumption that pups orient towards heat sources (thermotaxis), with the addition of heat generation, decay, and exchange accounting for the emergence of large stable huddles, and with the decay of individuals’ heat towards a target temperature accounting for the continuous pup flow (Figs 4–6).

According to the model, at low ambient temperatures agents orient towards littermates, which increases contacts and thus increases the exchange of heat between littermates. In simulations where body temperatures are held constant at 37°C this leads to weak huddling, sustaining only relatively small aggregates of *N* ≤ 4 pups. When the ambient temperature increases, the behaviour of the individuals effectively switches, and the same underlying mechanism instead causes littermates to orient away from contacts, causing the huddle to dissipate, and thus accounting for the phase transition measured by Canals et al. [9]. The addition of body heat decay stabilises the dynamics and thus enables a much larger huddle of *N* > 6 pups to be maintained. In larger huddles, pups closer to the centre have less exposed surface area than those at the periphery, which leads to a more dynamic exchange of positions. During strong huddling we observe the relative positions of the littermates to be fluid, with individuals cycling between the periphery and the center. In contrast, during weak huddling, we noted that while the distance of each individual from the center of the huddle remained fairly constant, there was a tendency for the overall center of mass of the huddle to drift and for its shape to skew.

Groups of eight 7-day or 10-day old rat pups have been observed to aggregate, on average, in around 3 or 1.5 subgroups, respectively [28, 29, 30]. Interestingly, for 10-day olds, Schank & Alberts [28] report a distribution of subgroups similar in form to that which we might expect if subgroups formed randomly, at a constant rate (see supplementary material S1 Text), but with a smaller mode of around 2 subgroups; see figure 7a of [28]). These experiments were conducted at warm ambient temperatures of 34°C, where our model would predict the average number of subgroups to be much larger (Fig 5B).

This apparent discrepancy may be accounted for by the size (and to a lesser extent the shape) of the arena boundary. We modelled a large circular arena like that of [9] and found that this has little influence on the group behaviour; simulations where we remove boundary interactions (i.e., by setting *β*_*i*_ = 0; see Models) produce no noticeable differences in any of our group-level metrics, except for a negligible reduction in *A*_min_. However, the experiments of [28, 29, 30] were conducted in much smaller rectangular arenas in order to investigate the effects of pup-pup versus pup-arena interaction. A physical model of huddling, using pup-shaped robots that move according to random control strategies, showed that in arenas of equivalent shape and size, aggregation levels comparable with those of real rat pups (averaging around 2.5 subgroups) can emerge [29]. As the individual-level control was essentially random, these robot experiments could not account for the temperature-dependent huddling effects measured by [9] and reproduced by our model, however they demonstrate an important role for agent-environment interaction in governing the dynamics of group behaviour. It would be interesting to test whether the larger numbers of subgroups predicted by our model emerge in similar experiments with pups when the arena size is increased.

Although our simulations show how the huddle, once formed, can be maintained by local thermotaxic interactions, it is important to note that we essentially pre-formed macro-huddles at the beginning of each simulation to avoid the appearance of locally stable micro-huddles, and that this may be considered to be a crude form of the global supervisory mechanism that we have claimed that the model does not require. When agents instead begin randomly distributed across the entire arena, it becomes increasingly unlikely that a single huddle will emerge. The endothermic model may be correct in predicting that micro-huddles are a more stable solution than the macro-huddle, which are unlikely to reform once the pups are dispersed, particularly in a large arena and in the absence of the dam who is otherwise known to herd isolated pups back towards the huddle [31, 32]. An extension to the model that could increase the tendency for macro-huddles to reform once dispersed could allow pups to respond to thermotactile cues sensed more distally, perhaps by sensing a temperature gradient radiating from the position of each littermate. However we note that for the experiment of [9], which first quantified the phase transition and first inspired our modelling approach, the arena was carefully ventilated so as to precisely control the ambient temperature and thus to minimise heat diffusion. We might similarly appeal to alternative forms of distal communication between pups, such as olfactory sensing or vision, however during early postnatal development pups are known not to respond to such cues [8]. Note that adding noise to the movement of each pup might help overcome micro-huddling, but that we chose to avoid adding unnecessary non-linearities into the system, to avoid creating a smooth phase transition by arbitraily smoothing an underlying step-function (as demonstrated in Fig 3).

**Fig 8:**
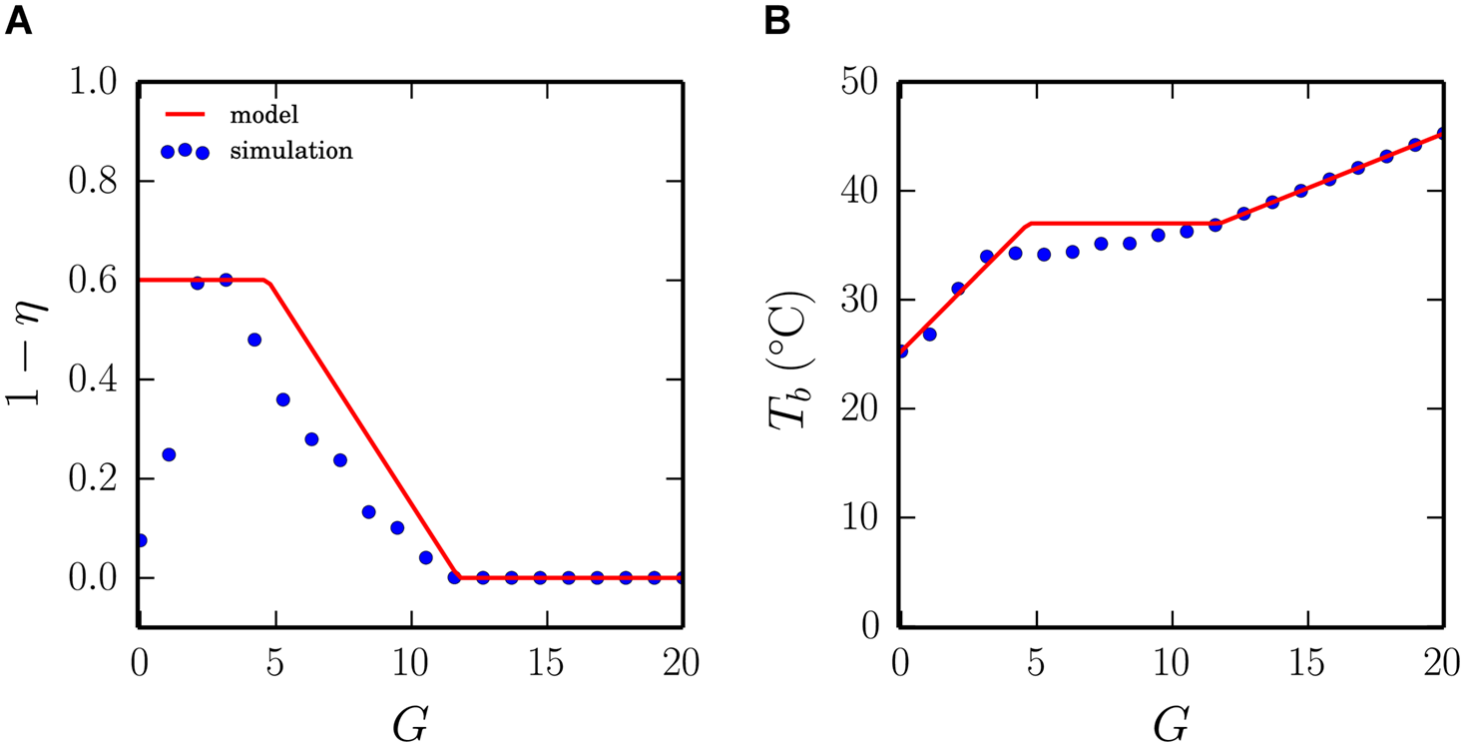
The role of thermogenesis. Intuitively, an animal that is able to produce more heat internally (e.g. through BAT-thermogenesis) could tolerate having a greater exposed surface area. Experiments have shown that when BAT-thermogenesis is pharmaceutically increased, rats will adapt to balance behavioural thermoregulation with the altered internal state. However, it has also been shown that animals without BAT-thermogenesis (Syrian golden hamsters) and rats with BAT-thermogenesis pharmaceutically inhibited will not display huddling behaviour. We tested the effects of varying the thermogenesis term *G* in the model and found the same pattern (*T*_*a*_ = 25.26). (A) At very low values of *G* contacts cannot be reliably maintained and huddling ceases at *G* = 0. As *G* increases, body temperatures become larger than the ambient temperature and macro huddling occurs (rising phase). As *G* further increases huddling is maximum within geometrical constraints (plateau phase). Increasing *G* further reduces the degree of huddling such that the collective behaviour maintains the group at their preferred body temperature *T*_*p*_ (falling phase). (B) Self-organised huddling is able to maintain the average body temperature of the group across a wide range of thermogenic rates.

Our model predicts that increasing thermogenesis will increase the critical temperature for the emergence of huddling (see Fig 7). In line with this prediction, recent theories suggest that rather than constituting a separate mechanism to huddling, thermogenesis is a necessary prerequisite for the emergence of thermoregulatory huddling. This is evidenced by data from experiments with Syrian golden hamsters, who do not huddle before brown adipose tissue (BAT) thermogenesis comes online at around postnatal day 14 [33], and with rats who cease to huddle when BAT is pharmaceutically blocked [34]. Furthermore, groups of rats comprising more BAT-disabled individuals lose their heat more rapidly [22], and when non-huddling hamsters are introduced into rat litters they begin to exhibit huddling behaviours [33]. Hence individual thermogenesis appears to be an essential ingredient for thermoregulatory huddling to emerge. Consistent with this view, setting the thermogenesis term *G* to 0 in the model disables huddling altogether and causes body temperatures to decay rapidly to the ambient temperature. Fig 8 shows the results of varying *G* in the homeothermotaxic individuals model, and reveals how the model can account for each of these data points.

The model thus accounts for the integral role of thermogenesis in huddling, because a key requirement of the model is that each pup acts as a thermogenic heat source to direct the thermotaxic movement of its littermates. The model accounts for the role of thermogenesis as a crucial source of heterogeneity amongst individual body temperatures that is required for temperature-dependent dynamic group behaviours to emerge. Hence, thermogenesis provides the energy source and fulfils the symmetry-breaking requirements for the emergence of huddling. Our modelling results therefore suggest that thermogenesis does not cause huddling *per se*, but rather allows huddling to reveal itself in groups of individuals that orient towards preferred temperatures. We can thus describe the emergence of huddling as the natural expression of collective thermotaxis by thermogenic individuals.

## Models

At a given simulation time the components that effect the body temperature of each pup are determined as follows. The location of the center of pup *i* in the two-dimensional arena is given by the coordinate **x**_*i*_ = (*x*_*i*_, *y*_*i*_), and its orientation is defined as *θ*_*i*_. We index the littermates of pup *i* by *j* ≠ *i*, and we index *n* = 1000 thermometers tiling the circumference of its circular body by *k*. Thermometers are located at coordinates $\left(x_{ik}=x_{i}+r\;\text{cos}\left(\theta_{i}+(k-\frac{1}{2})\frac{2\pi }{n}\right),y_{ik}=y_{i}+r\;\text{sin}\left(\theta_{i}+(k-\frac{1}{2})\frac{2\pi }{n}\right)\right)$, which means that the first *n*/2 = 500 thermometers are on the left of pup *i* and the second half are on its right. The radius *r* is common to all pups. To determine the heat transferred via surface contacts for each pup *i*, we first decide whether a given thermometer *k* falls within the boundary of each pup *j*, and record the result as *α*_*ijk*_,

$$\begin{array}{c} \alpha_{ijk}=\{\begin{array}{cc} 1 & \text{if}\;d_{ij}\leq 2r\;\wedge \;[\theta_{i}+\left(k-\frac{1}{2}\right)\frac{2\pi }{n}-\phi_{ij}]<\cos^{-1}\frac{d_{ij}}{2r}\\ 0 & \text{otherwise}, \end{array} \end{array}$$ (5)

where *ϕ*_*ij*_ = arctan2(*y*_*j*_−*y*_*i*_, *x*_*j*_ − *x*_*i*_) and $d_{ij}=\sqrt{{\left(x_{j}-x_{i}\right)}^{2}+{\left(y_{j}-y_{i}\right)}^{2}}$ give the angle and distance of pup *j* from pup *i*, and [.] = *π*−∣*π*−∣.∣∣ denotes absolute distance around the circle. We can therefore determine that thermometer *k* is exposed if *α*_*ijk*_ is zero for all *j*, using *ϵ*_*ik*_ = ∏_*j*_(1 − *α*_*ijk*_), because the product will be 1 only when thermometer *k* falls outside of the boundary of each of the other pups. This in turn allows us to define the proportion of the surface area of the pup that is exposed to be $\eta_{i}=\frac{1}{n}\sum_{k}\epsilon_{ik}$. The temperature at a thermometer in contact with other pups is $\chi_{ik}=\alpha_{ij^{\prime }k}T_{b}^{j^{\prime }}$, where *T*_*b*_ is body temperature, and *j*′ = argmin_*j*_((*x*_*j*_ − *x*_*ik*_)^2^+(*y*_*j*_ − *y*_*ik*_)^2^) indexes the littermate that is closest to thermometer *k*. Thus we can define the contact-mediated surface temperature to be $T_{c}^{i}=\frac{1-\eta_{i}}{n}\sum_{k}\chi_{ik}$. The terms *η*_*i*_ and $T_{c}^{i}$ are used for the body temperature update equation, which is reproduced here for completeness,

$$\begin{array}{c} \frac{dT_{b}^{i}}{dt}=-k_{1}\eta_{i}\left(T_{b}^{i}-T_{a}\right)-k_{2}\left(1-\eta_{i}\right)\left(T_{b}^{i}-T_{c}^{i}\right)+G, \end{array}$$ (6)

where *k*_1_ and *k*_2_ are thermal conductance constants for exposed and contact regions respectively, *G* is the rate of thermogenesis, and *T*_*a*_ is the ambient temperature.

The kinematics of each pup is driven by the difference between the average surface temperature on its left and right. The temperature at each thermometer is *τ*_*ik*_ = *T*_*a*_ *ϵ*_*ik*_ + *χ*_*ik*_, and the surface temperature on its left and right can be defined as,

$$\begin{array}{c} T_{L}^{i}=\frac{2}{n}\sum_{k=1}^{n/2}\tau_{ik},\;T_{R}^{i}=\frac{2}{n}\sum_{k=n/2+1}^{n}\tau_{ik}. \end{array}$$ (7)

For the homeothermotaxic model only we apply a function to $T_{L}^{i}$ and $T_{R}^{i}$ before motor speeds are calculated such that $F(T_{L}^{i})={\left(1+e^{-\frac{1}{\sigma }\left(T_{p}-T_{b}^{i}\right)T_{L}^{i}}\right)}^{-1}$ and similarly for $T_{R}^{i}$, where *T*_*p*_ is the preferred temperature. For all but the homeothermotaxic model we use simply $F(T_{L}^{i})=T_{L}^{i}$.

Thermotaxic orienting is based on the difference between $T_{L}^{i}$ and $T_{R}^{i}$. These sensor values determine motor speeds $M_{L}^{i}=\frac{F(T_{R}^{i})}{F(T_{L}^{i})+F(T_{R}^{i})}$ and $M_{R}^{i}=\frac{F(T_{L}^{i})}{F(T_{L}^{i})+F(T_{R}^{i})}$, which in turn are used to determine the rate of change in orientation. On each timestep, each pup is rotated by,

$$\begin{array}{c} \frac{d\theta_{i}}{dt}=\tan^{-1}(v_{1}\left(M_{L}^{i}-M_{R}^{i}\right)), \end{array}$$ (8)

and translated at,

$$\begin{array}{c} \frac{dx_{i}}{dt}=v_{2}(\;\begin{array}{c} \cos\theta_{i}\\ \sin\theta_{i} \end{array}\;)+\beta_{i}\frac{x_{i}}{|x_{i}|}+\sum_{\left\{j:d_{ij}\leq 2r\right\}}(r-\frac{d_{ij}}{2})\frac{x_{i}-x_{j}}{|x_{i}-x_{j}|}, \end{array}$$ (9)

where *v*_1_ and *v*_2_ control the speed of rotation and forward motion, respectively. A circular arena boundary of radius *r*_arena_ = 10*r* centered at the origin is enforced by defining *β*_*i*_ = (*r*_arena_−∣**x**_*i*_∣−*r*) if ∣**x**_*i*_∣+*r* ≥ *r*_arena_, else *β*_*i*_ = 0. Collisions between pups are resolved via the final term in Eq 9, which ensures that pup *i* is pushed directly away from each pup with which it makes contact, with a force that increases with the degree of overlap between them. Note that because the translation speed *v*_2_ is constant, Eq 9 ensures that unobstructed pups will always be moving, hence the model does not attempt to capture any relationship between the temperature of a pup and its activity, which may play a role in rodent huddling.

Pups are initialised at uniform random locations in a circle of radius *r*, with random orientations *θ*, and an initial *T*_*b*_ = 30°C is allowed to settle for 100 timesteps before kinematics are enabled. Unless otherwise stated, the following parameters were used for all simulations reported: $k_{1}=\frac{1}{2\pi r}$, $k_{2}=\frac{2.5}{2\pi r}$, *v*_1_ = 200, *v*_2_ = 0.3, *G* = 6.32, *T*_p_ = 37°C, *σ* = 100, and *dt* = 0.05. All simulations were implemented using the python programming language (using the NumPy and SciPy packages); an implementation of the model is provided as supplementary material S1 File.

## Supporting Information

S1 Text Supplementary analysis of micro-huddling. Using a combinatorial approach, we establish a baseline level of expected aggregation, under the assumption that micro-huddles form randomly at a constant rate. The approach is based on integer partitioning, for which an algorithm is provided.
(PDF)

S1 File Implementation of the homeothermotaxic model. Written in optimized python code (requires NumPy and SciPy packages). The script returns the average body temperature and value of the huddling metric (1 − *η*) over ten repeated experiments at ambient temperature 25°*C*, with thermogenesis *G* = 6.32, i.e., returns a data point equivalent to that reported in Fig 4.
(ZIP)
