# Supplementary material for: A Self-Organising Model of Thermoregulatory Huddling
Source: PLoS Comput Biol. 2015 Sep 3;11(9):e1004283. doi: 10.1371/journal.pcbi.1004283 (PMC4559402; doi:10.1371/journal.pcbi.1004283)
Supplement: S1 Text — Using a combinatorial approach, we establish a baseline level of expected aggregation, under the assumption that micro-huddles form randomly at a constant rate. The approach is based on integer partitioning, for which an algorithm is provided. (PDF) [file pcbi.1004283.s001.pdf]

A Self-Organising Model of Thermoregulatory Huddling

Jonathan Glancy, Roderich Groß, James V. Stone, Stuart P. Wilson

Supplementary Analysis of Micro-Huddling

To explore the relationship between pup flow and the exchange of pups between micro-huddle states further, we sought to define a baseline aggregation level, against which huddling at different ambient temperatures could be compared. To this end we first note that a group of N pups may be grouped in many different ways. For example, we might observe $N = 8$ pups grouped in three subgroups comprising 4, 3, and 1 contacting pups, or in four subgroups comprising 4, 2, 1, and 1 pups, or in four subgroups of 3, 3, 1, and 1 pups etc. Schank & Alberts refer to these patterns, e.g., (4,3,1), (4,2,1,1), (3,3,1,1), as 'aggregons' [1], and show that for $N = 8$ pups there are 22 unique aggregons including the macro-huddle (8) and no-huddle (1,1,1,1,1,1,1,1) aggregons. For $N = 8$ pups, the number of aggregons comprising $H = \{1, 2, 3, 4, 5, 6, 7, 8\}$ subgroups are $\{1, 4, 5, 5, 3, 2, 1, 1\}$ (see table 1 of [2]), and dividing these frequencies by the total defines a probability mass distribution $p(H) = \{\frac{1}{22}, \frac{4}{22}, \frac{5}{22}, \frac{5}{22}, \frac{3}{22}, \frac{2}{22}, \frac{1}{22}, \frac{1}{22}\}$. For eight pups randomly forming new aggregons at a constant rate, the average number of subgroups is therefore the expected value of $p(H)$, which is $E(H) = \sum_{i=1}^{N=8} p(h_i)h_i = 3.91$ subgroups. In more general terms, the unique aggregons for a given N are referred to as the integer partition of N [3], and in Figure 1 of this document we present a recursive algorithm for partitioning N, which calculates the unique aggregons, $p(H)$ and $E(H)$. For our $N = 12$ pup simulations the algorithm returns $p(H) = \{\frac{1}{77}, \frac{6}{77}, \frac{12}{77}, \frac{15}{77}, \frac{13}{77}, \frac{11}{77}, \frac{7}{77}, \frac{5}{77}, \frac{3}{77}, \frac{2}{77}, \frac{1}{77}, \frac{1}{77}\}$ and thus predicts an average of $E(H) = 5.18$ subgroups. In Fig. 5B of the main text we show how the average number of subgroups in our simulations varies with the ambient temperature. The relationship follows an approximately sigmoidal curve with the theoretical 5.18 subgroups average occuring at an ambient temperature of $\approx 25°$C, close to the middle of the linear zone of the sigmoid where we indeed expect pups to be flowing between subgroup states.

Comparing the average number of subgroups with the theoretical baseline $E(H)$ may therefore be a useful way of relating pup flow to aggregation levels, and for comparing experimental data with the group behaviour predicted by the model. For further discussion of the validity of this combinatorial approach see [4, 5].

References

[1] Schank JC, Alberts JR (1997) Self-organized huddles of rat pups modeled by simple rules of individual behavior. Journal of Theoretical Biology 189: 11–25.

[2] May CJ, Schank JC, Joshi S, Tran J, Taylor RJ, et al. (2006) Rat pups and random robots generate similar self-organized and intentional behavior. Complexity 12: 53–66.

[3] Andrews GE (1984) The theory of partitions. Cambridge University Press.

[4] Schank JC (1997) Problems with dimensionless measurement models of synchrony in biological systems. Am J Primatol 41: 65–85.

[5] Schank JC (2004) Avoiding synchrony as a strategy of female mate choice. Nonlinear Dynamics Psychol Life Sci 8: 147–176.

```
def Split(C,A,S):
    # A: parts already split
    # C: part to split
    # S: number of splits
    for B in range(1,C):
        #split C into new parts B and C
        C-=1
        P = A+[B,C]
        #check no decreasing parts sizes in P
        up = True
        for i in range(1,S+1):
            if (P[i]>P[i-1]):
                up = False
                break
        #if not, count P as a unique partition
        if up:
            global H
            H[S]+=1
            print P
        #recursively split part C
        Split(C,A+[B],S+1)

def Partition(N):
    #initialize frequency histogram
    global H
    H = [1]
    for i in range(1,N):
        H+=[0]
    #begin recursive partition
    Split(N,[],1)
    print [N]
    #count total partitions
    T=0.
    for i in range(N):
        T+=H[i]
    #probability mass distribution p(H)
    pH=H
    for i in range(N):
        pH[i]=H[i]/T
    del(H)
    #calculate expected value of p(H)
    E=0.
    for i in range(N):
        E+=(i+1.)*pH[i]
    return pH,E,T

#partition N=8 pups
N=8
print 'aggregons: '
h,E,T = Partition(N)
print 'E(H)= '+str(E)
```

Figure 1. Python code for computing the integer partition of N. Prints the unique aggregon sequences and returns the probability mass distribution $p(H)$ and the expected value $E(H)$ for a given huddle size N.
